# Supplementary material for: Wavelength-multiplexed hook nanoantennas for machine learning enabled mid-infrared spectroscopy
Source: Nat Commun. 2022 Jul 5;13:3859. doi: 10.1038/s41467-022-31520-z (PMC9256719; doi:10.1038/s41467-022-31520-z)
Supplement: Supplementary file 1 — Supplementary information [file 41467_2022_31520_MOESM1_ESM.pdf]

Supplementary Information –

Wavelength-Multiplexed Hook Nanoantennas for Machine Learning Enabled Mid-IR Spectroscopy

Zhihao Ren[1,2,3,5], Zixuan Zhang[1,2,3,5], Jingxuan Wei[1,2,3], Bowei Dong[1,2,3], Chengkuo Lee[1,2,3,4*]

[1] Department of Electrical and Computer Engineering, National University of Singapore, Singapore 117583, Singapore.

[2] Center for Intelligent Sensors and MEMS (CISM), National University of Singapore, Singapore 117583, Singapore.

[3] NUS Suzhou Research Institute (NUSRI), Suzhou, Jiangsu 215123, China.

[4] NUS Graduate School - Integrative Sciences and Engineering Programme (ISEP), National University of Singapore, Singapore 119077, Singapore.

[5] These authors contributed equally: Zhihao Ren, Zixuan Zhang

*E-mail: elelc@nus.edu.sg

Note S1: Benchmark of hook nanoantennas

Table S1. The benchmark table of MIR nanoantenna sensors

Sensing unit	Optimization methods	Critical dimension	Field enhancement	Sensing analytes	Wavenumber (cm^{-1})	SEIRA Sensing Signal (%)
Bowtie nanoantenna[1]	Nanogap	Sub-3 nm gap	10^7	4-NTP	1515, 1335	0.003% for monolayer
				4-MTP	1594, 1485	0.008% for monolayer
Coaxial Zero-Mode Resonators[2]	Nanogap	7 nm	10^6	Silk	1668, 1532	378 mOD for 5nm thin-film
Graphene Nanoribbon[3]	Field confinement by 2D materials	~3 nm thickness	10^4	Protein	1668, 1532	27% for bilayer
Hybrid Graphene Nanorod[4]	Field confinement by 2D materials	10 nm gap	5×10^4	Glucose	1470	2% for 100 pmol
Nanorod MA[5]	Hotspot release, Nanogap	30 nm vertical gap	10^9	H_2O, Ethanol, Acetone	3390	0.8364%/pM
Cross MA[6]	Hotspot release, Nanogap	10 nm vertical gap	8×10^8	ODT	2850-2995	36% for 2.8 nm thickness
Nanorod[7]	Nanogap, Molecules Enrichment	80 nm gap	10^5	Lipids	2850, 2920	10 mOD for bilayer
				Melittin	1650, 1550	10 mOD for 6×10^{-6} M
				Sucrose	1142	20 mOD for 200 mg/mL
				Nucleotides	1230	20 mOD for 200 mg/ML
Nanograting MA[8]	Hotspot release, Molecules Enrichment	200 nm vertical gap	1600	Proline	1400, 1600	10% for 0.2 µg/ml,
				D-glucose	1400, 1640	5% for 0.1 µg/ml
Nanorod[9]	Molecules Enrichment	~100 nm gap	10^5	Benzene(gas)	1480	4×10^{-4} OD for 25 ppb
Cross MA[10]	Molecules Enrichment	200 nm vertical gap	2500	CO_2	2347	0.0358%/ppm
				CH_4	1305	0.0121%/ppm
Cross MA[11]	Molecules Enrichment	200 nm vertical gap	4900	PECA	1740, 1200	15% for 10 nm thickness
Nanodisk MA[12]	Molecules Enrichment	200 nm vertical gap	1000	miR-155	1700	0.42% for 500 pM
WMHNA	Loss engineering	~200 nm width	10^5	PMMA	1750, 1470, 1180	16% for 20nm
				Silk	1668, 1532, 1200	50% for 50nm
				Methanol	1540-1300	5.18% for 1%
				Ethanol	1540-1170	3% for 1%
				Isopropanol	1540-1120	4.74% for 1%

*MA: Metamaterial Absorber, consisting of metal-isolator-metal (MIM) structure.

Since many previous works were designed at different wavelengths for different sensing analytes, it is difficult to tell the sensing performance among different works. Therefore, we perform a comparison among the nanoantenna designs in previous works and our HNA devices. As shown in **Fig. S1a**, we choose two control designs from previous papers about the standard Fano-resonant structure (Fano-resonant asymmetric metamaterial, FRAMM)[13] and dipolar resonant structure (Nanorod antenna, NA)[14]. To make a fair comparison, we match the resonance wavelength of all devices to the absorption peak of carbonyl stretching from poly (methyl methacrylate) (PMMA) at the 5.8 μm wavelength. A scaling factor (S) is applied to all geometric parameters of the control devices to tune the resonance frequencies from the original position (in the previous works) to 5.8 μm wavelength. The thickness of all antenna devices and PMMA thin film are fixed at 70 nm and 10 nm, respectively. The detailed geometric parameters are shown in Table S2. In Lumerical FDTD solver, we build up a Lorentz oscillator model of carbonyl stretching resonance from PMMA. The complex relative permittivity as a function of resonance frequency (*f*) is described as

$$\varepsilon(f) = \varepsilon_0 + \frac{\varepsilon_{Lorentz}\omega_0^2}{\omega_0^2 - 4\pi i \delta_0 f - (2\pi f)^2} \tag{S1}$$

where background permittivity (ε_0), Lorentz permittivity ($\varepsilon_{Lorentz}$), Lorentz resonance (ω_0), and Lorentz linewidth (δ_0) are set to be 1, 0.002, 3.25×10^{14} rad/s, and 1.5×10^{12} rad/s, respectively. The sensing results of different devices are shown in **Fig. S1b, d**. The extracted spectrum difference is shown in **Fig. S1c, e**, representing the sensing performance of each device. The normalized sensitivity of each device is shown in **Fig. S1f.** In reflection mode, the optimized HNA-1 device improves the sensitivity by 28.2% and 83.6% compared to FRAMM and NA, respectively. In transmission mode, the enhancement of 2.41 and 17.28 times of sensitivity is demonstrated by the optimized HNA-2 device compared with FRAMM and NA, respectively. From the obvious improvement above, the HNA device is a good candidate for ultrasensitive MIR molecular sensing.

The sensing performance can be optimized from the radiative loss of HNA by tuning the folding degree of the HNA structure (ΔL, defined by L_1-L_3). According to the optimal loss rates at transmission (*f=0.5*) and reflection(*f=2*) modes, two HNA devices with different ΔL are designed as optimized structures for transmission (HNA-2) and reflection (HNA-1) modes. Because the *f* difference between HNA-2 to control devices is larger than the *f* difference between HNA-1 to control devices, the improvement of sensitivity in transmission mode is achieved much larger than that in reflection mode (241% to 28.2% for FRAMM, 1728% to 83.6% for NA).

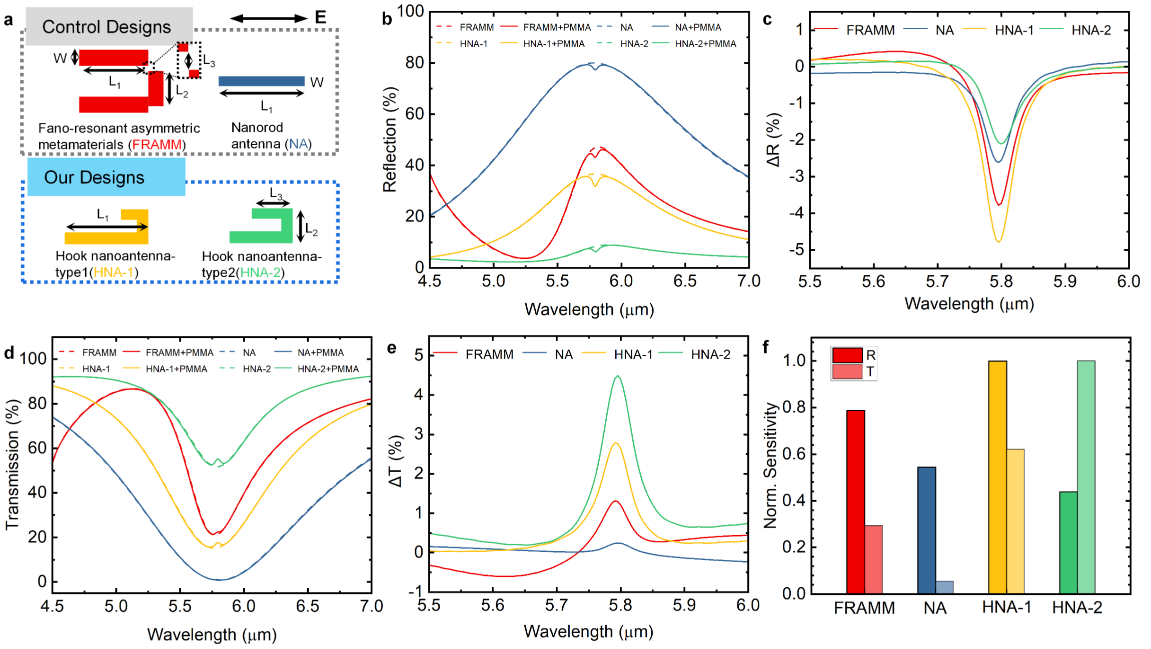

Fig. S1 Sensing performance of different devices. **a** Schematic drawing of hook nanoantenna (HNA) devices and control devices, including Fano-resonant asymmetric metamaterials (FRAMM)[13] and nanorod antenna (NA)[14]. **b** The simulated reflection spectrum of all nanoantenna devices with thin-film analytes of poly (methyl methacrylate) (PMMA). All devices are designed to match with the absorption peak of carbonyl stretching at 5.8 μm wavelength. **c** The extracted reflection difference (ΔR) of different devices. **d** Simulated transmission spectrum of all nanoantenna devices with thin-film analytes of PMMA. **e** The extracted transmission difference (ΔR) of different devices. **f** Normalized sensitivity of different devices for thin-film sensing. HNA devices perform the best sensitivity in reflection mode (HNA-1) and transmission mode (HNA-2).

Table S2. The geometric parameters of control and HNA devices

Devices	L_1 (nm)	L_2 (nm)	L_3(nm)	W(nm)	P_x(nm)	P_y(nm)	T (nm)	S
FRAMM	1350	680	220	270	3200	2700	70	0.75
NA	2180	N.A.	N.A.	198	2723	2723	70	0.99
HNA-1	1320	380	500	100	2950	2500	70	N.A.
HNA-2	1200	380	700	100	2700	2500	70	N.A.

*L_1, L_2, L_3, W: labeled in Fig. S1. P_x, P_y: the period in the horizontal and vertical direction in Fig. S1. T: the thickness of antennas. S: scaling factor, the ratio between our parameters and original ones in previous papers.

Note S2: Theoretical analysis of nanoantenna sensor using TCMT

The temporal coupled-mode theory (TCMT)[15] is used to describe the coupling behavior between PNA and molecular vibration. We treat the plasmonic resonance (denoted as P) as a bright mode that is coupled to the incident light, while we treat the molecular vibration (denoted as M) as a dark mode, in which coupling efficiency is much lower than PNA and can be ignored in their coupling system. Therefore, we obtain the equations using TCMT as

$$\frac{dP}{dt} = j\omega_0 P - (\gamma_a + \gamma_r)P + j\mu M + \sqrt{\gamma_r} S_{in} \tag{S2}$$

$$\frac{dM}{dt} = j\omega_m M - \gamma_m M + j\mu P \tag{S3}$$

$$S_t = S_{in} - \sqrt{\gamma_r} P \tag{S4}$$

$$S_r = \sqrt{\gamma_r} P \tag{S5}$$

where P, M and ω_0, ω_m represent the amplitude and angular frequency of resonance for HNA and molecular vibration, respectively. γ_a and γ_r denote the radiative and absorptive losses of HNA, while γ_m is the absorptive loss of molecules. μ is the coupling strength between HNA and molecular vibration. S_{in}, S_t, *and* S_r represent the amplitude of incident, transmitted, and reflected light, respectively. Under the time-harmonic condition, the first derivative of time (*d/dt*) is replaced by $j\omega$, and the Equation S2 and Equation S3 can be simplified as

$$[j(\omega - \omega_0) + (\gamma_a + \gamma_r)]P = j\mu M + \sqrt{\gamma_r} S_{in} \tag{S6}$$

$$[j(\omega - \omega_0) + \gamma_m]M = j\mu P. \tag{S7}$$

By substituting Equation S7 to Equation S6, we can eliminate M and obtain

$$[j(\omega - \omega_0) + (\gamma_a + \gamma_r) + \frac{\mu^2}{j(\omega - \omega_0) + \gamma_m}]P = \sqrt{\gamma_r} S_{in} \tag{S8}$$

Therefore, transmission and reflection can be expressed as

$$T(\omega) = \left|\frac{S_{\mathrm{t}}}{S_{\mathrm{in}}}\right|^2 = \left|\frac{j(\omega-\omega_0)+\gamma_a+\dfrac{\mu^2}{j(\omega-\omega_m)+\gamma_m}}{j(\omega-\omega_0)+(\gamma_a+\gamma_r)+\dfrac{\mu^2}{j(\omega-\omega_m)+\gamma_m}}\right|^2 \tag{S9}$$

$$R(\omega) = \left|\frac{S_{\mathrm{r}}}{S_{\mathrm{in}}}\right|^2 = \left|\frac{\gamma_r}{j(\omega-\omega_0)+(\gamma_a+\gamma_r)+\dfrac{\mu^2}{j(\omega-\omega_m)+\gamma_m}}\right|^2 \tag{S10}$$

The Fano-like line shape can be expressed from Equations S9, S10 for both transmission and reflection modes. The plasmonic resonance can be easily obtained when there is no coupling effect from molecules (μ=0).

$$T(\omega) = \frac{(\omega-\omega_0)^2+{\gamma_r}^2}{(\omega-\omega_0)^2+(\gamma_a+\gamma_r)^2} \tag{S11}$$

$$R(\omega) = \frac{{\gamma_a}^2}{(\omega-\omega_0)^2+(\gamma_a+\gamma_r)^2} \tag{S12}$$

$$A(\omega) = 1-T-R = \frac{2\gamma_a\gamma_r}{(\omega-\omega_0)^2+(\gamma_a+\gamma_r)^2} \tag{S13}$$

Equations S9, S10 are used to extract absorptive and radiative loss of HNA by fitting the resonance spectrum in the frequency domain from simulation (**Fig. S2**). By engineering the HNA structure by changing ΔL with the constant L, the γ_r and γ_a can be tuned continuously, and ω_0 remains unchanged. To explore the sensing performance, we have made some assumptions to simplify Equation S9 to perform the analytical operation. First, we make $\omega_0=\omega_m$ to match the frequency of HNA and molecular vibration since the WMHNA is only designed for the molecular absorption wavelength near the HNA resonance wavelength to have the best enhancement. Second, we treat μ as a much smaller parameter compared with γ_m, γ_r, and γ_a. Therefore, we apply the difference between Equation S10 and Equation S9 when $\omega = \omega_0 = \omega_m$.

$$\Delta T(\omega = \omega_0) = \frac{2\mu^2}{\gamma_m} \frac{\gamma_a \gamma_r}{(\gamma_a + \gamma_r)^2 (\gamma_a + \gamma_r + \frac{\mu^2}{\gamma_m})} + (\frac{\mu^2}{\gamma_m})^2 \frac{{\gamma_r}^2}{(\gamma_a + \gamma_r)^2 \left(\gamma_a + \gamma_r + \frac{\mu^2}{\gamma_m}\right)^2} \quad \text{(S14)}$$

Since $\mu << \gamma_m$, $\frac{\mu^2}{\gamma_m}$ is a small real number close to 0. Therefore, we cancel the high order term and simplify Equation S14 as

$$\Delta T(\omega = \omega_0) = \frac{2\mu^2}{\gamma_a \gamma_m} \frac{f}{(1+f)^3} \quad \text{(S15)}$$

where $f = \gamma_r / \gamma_a$, defining the ratio of radiative and absorptive loss. Similarly, for the reflection spectrum, we get

$$\Delta R(\omega = \omega_0) = -\frac{2\mu^2}{\gamma_a \gamma_m} \frac{f^2}{(1+f)^3} \quad \text{(S16)}$$

The negative sign in Equation S16 indicates the opposite change in transmission and reflection spectrum induced by molecular vibration. γ_a refers to the omics loss of material; thus, it is constant in our experiment of HNA made by Au. When changing ΔL of HNA, the electrical field does not change too much among different HNA devices, so that μ is also a constant. Additionally, γ_m is also unchanged since we fix the absorption peaks of the "C=O" bond from PMMA in sensitivity characterization. By applying the first derivative of f for Equation S15 and Equation S16. We further calculate the maximum enhancement of the T and R spectrum and get the optimal condition that occurs when f equals 0.5 and 2, respectively.

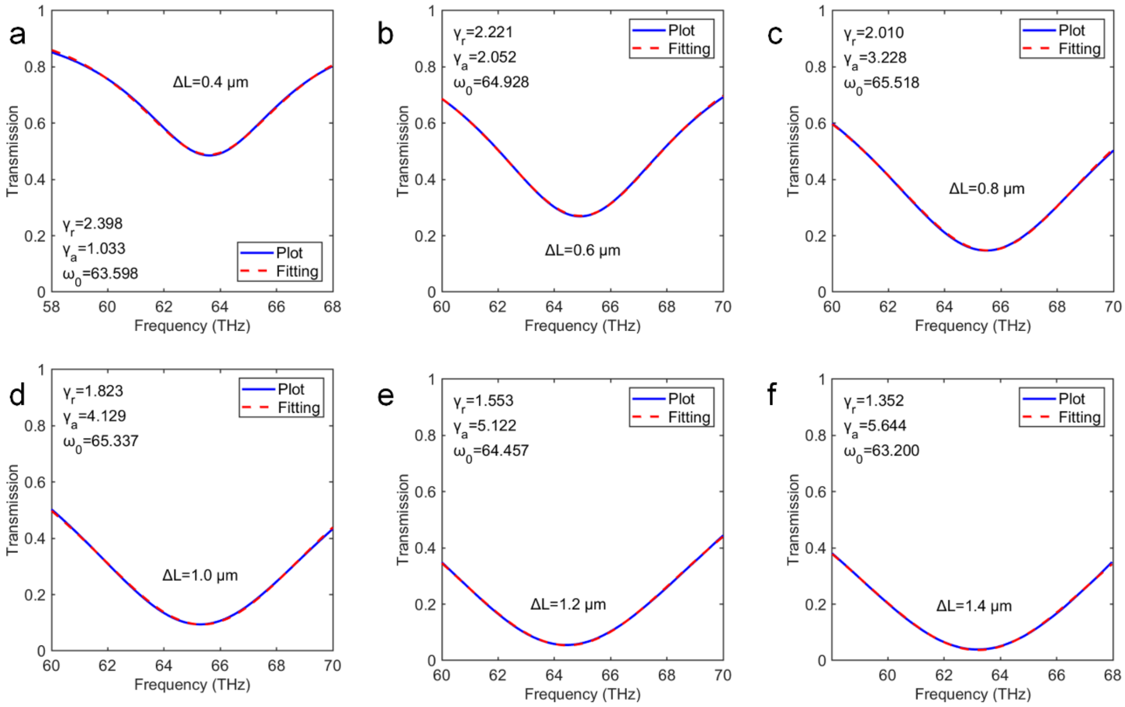

Fig. S2 The fitting curves of simulation results of hook nanoantennas at different ΔL to extract the theoretical parameter (γ_a and γ_r) in TCMT. The same fitting method is applied to experimental data for the discussion on sensing performance.

Note S3: Near-field distribution of HNA

To study the near field enhancement of HNA, we perform the FDTD simulation and monitor the electric field intensity and polarity at resonance wavelengths for different devices. The amplitude of the electric field of different devices is plotted in Fig. S2a using the log scale by the equation $\log(|E/E_0|^2)$. The results show that all of the devices have an enhanced field intensity at the magnitude of 10^5. The extracted field enhancement ($|E/E_0|^2$) at two arms of HNAs (position labeled in Fig. S2a) are shown in Fig. S2b. The normalized electric field of each device is characterized by E_x in Fig. S2c, showing a fundamental dipole mode of the resonance and the inverse current induced by the short arm of HNA to tune the radiative loss.

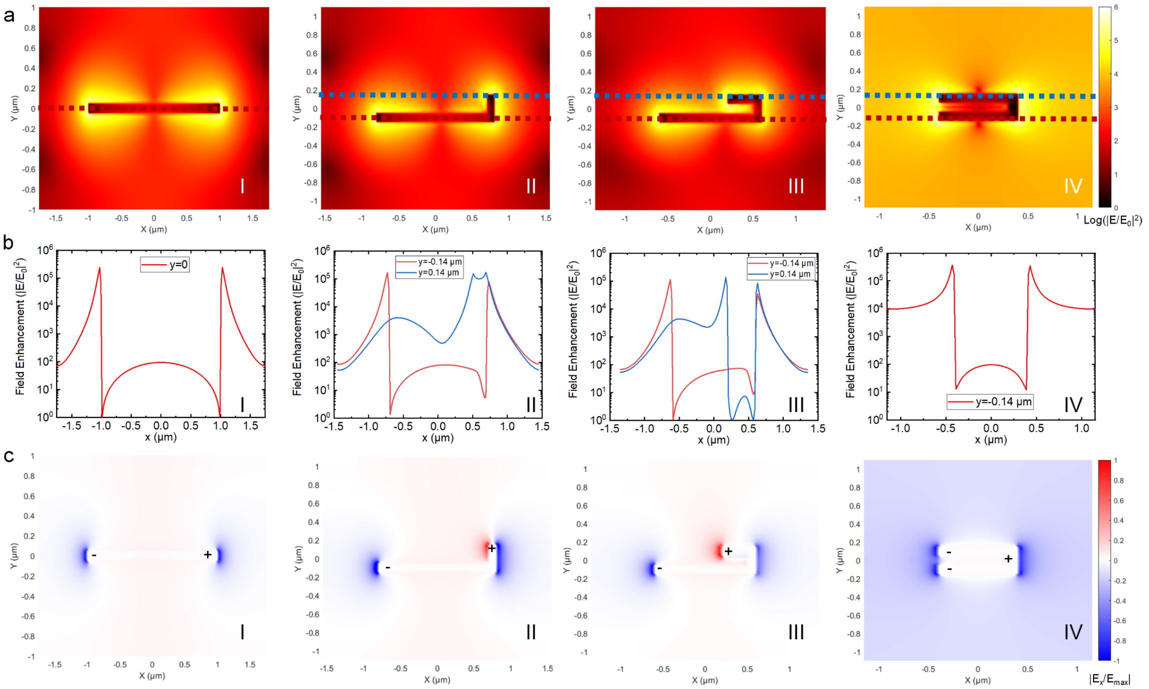

Fig. S3 The near-field distribution of HNA at different ΔL. **a** The enhancement of electric field intensity ($|E/E_0|^2$) of different HNAs. **b** The extracted field enhancement ($|E/E_0|^2$) at two arms of HNAs (position labeled in (a)). **C** The normalized electric field with the component at x-direction of different HNAs. The fundamental dipole mode is clearly shown from the polarity of the electric field (except for the extreme case of ΔL=0 in (IV)).

Note S4: Far-field characterization of HNA

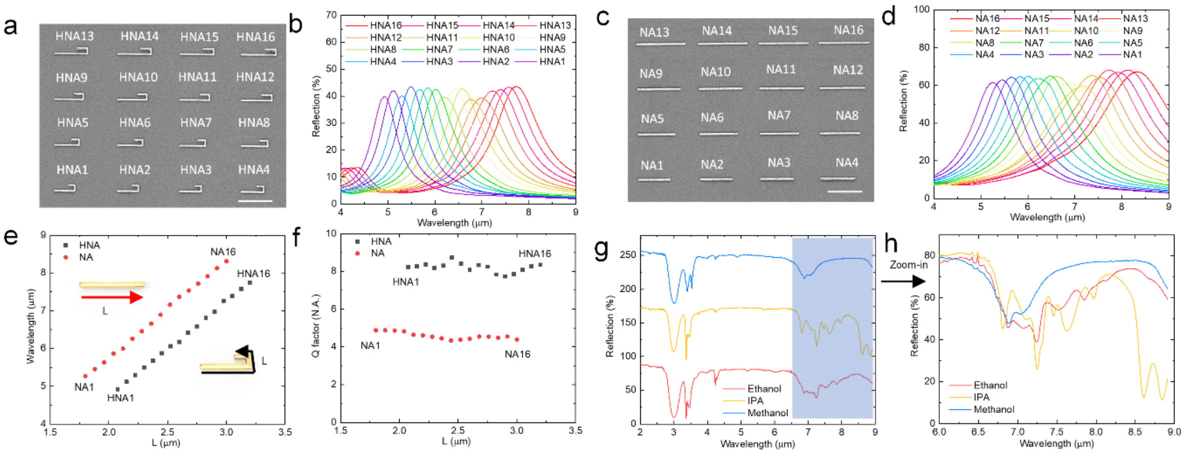

Fig. S4 a The SEM image of HNA supercell. The scale bar indicates 2 µm in length. **b** The far-field spectra of the antenna elements of HNA1 to HNA 16 tested by FTIR. **c** The SEM image of nanorod antenna (NA) supercell. The scale bar indicates 2 µm in length. **d** the far-field spectra of the antenna elements of NA1 to NA16 tested by FTIR. **e,f** The extracted resonance wavelength and quality factor of HNA1 to HNA16 and NA1 to NA 6. **g,h** The fingerprint absorption spectra of methanol, ethanol, and isopropanol liquid in 100% concentration, showing overlapping of the absorption peaks in broadband ranges.

Note S5: Size characterization of fabricated devices

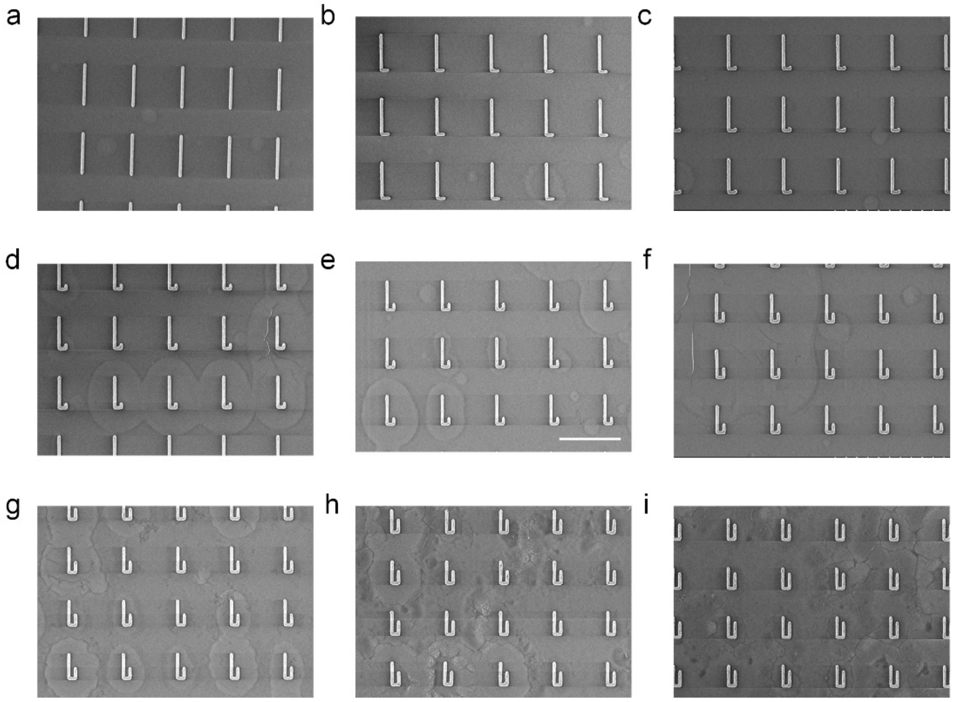

Fig. S5 The SEM image of HNA devices with different ΔL. The scale bar indicates 2 μm in length.

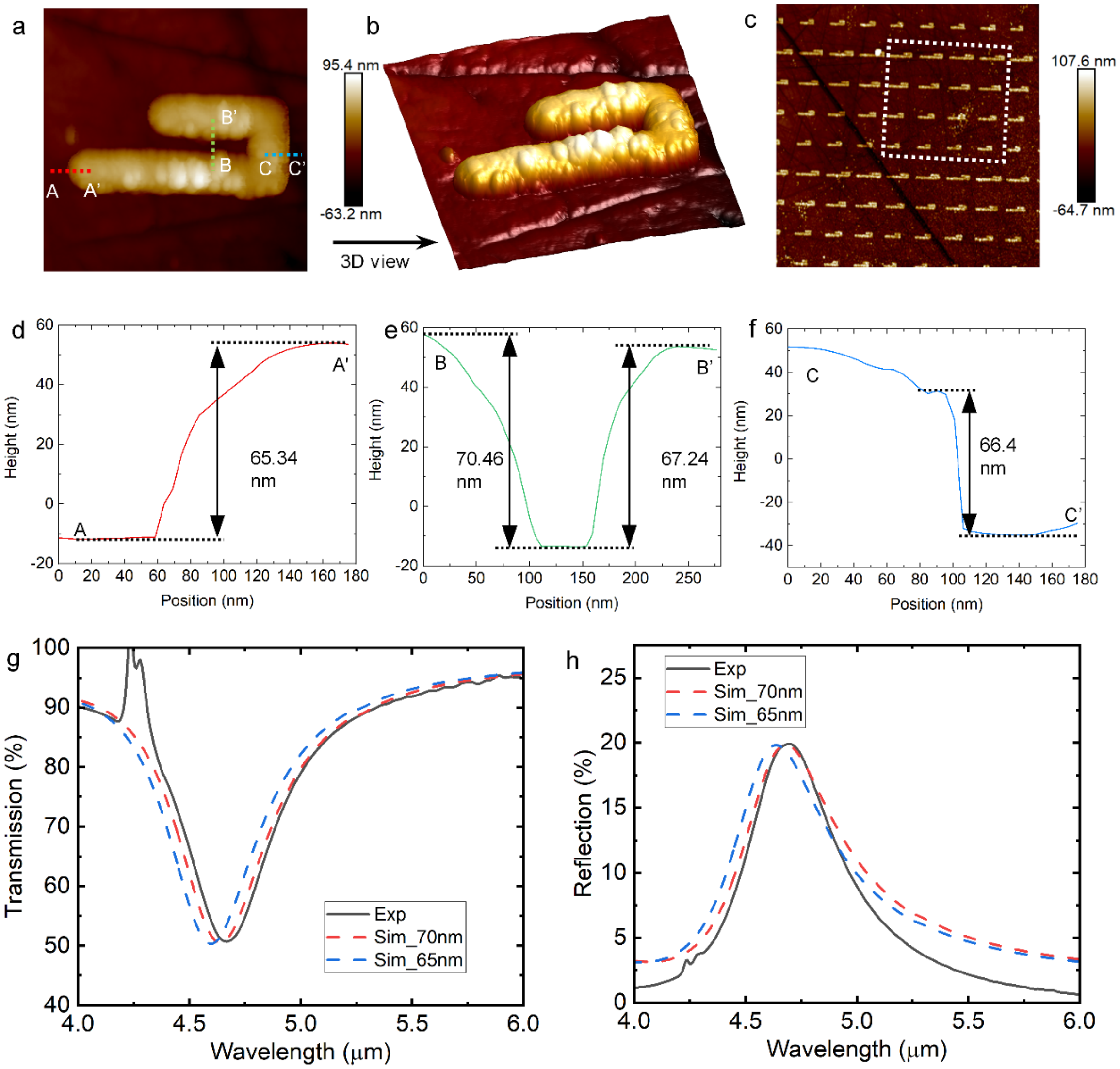

Fig. S6 a The AFM image of one HNA device. **b** The reconstructed 3D model of HNA from the height measurement in (**a**). **c** The AFM image of WMHNA with a highlight area of one unit cell. **d-f** The height measurement results of three parts of HNA, showing a range of antenna thickness is 65-70 nm. This is caused by the non-uniformity during the gold deposition. **g,h** Simulation and experiment results of transmission (**g**) and reflection (**h**) spectrum of HNA showing in (**a**). The different antenna thicknesses of 65 nm and 70 nm are simulated to compare with the FTIR measurement.

Note S6: Sensing characterization using different devices

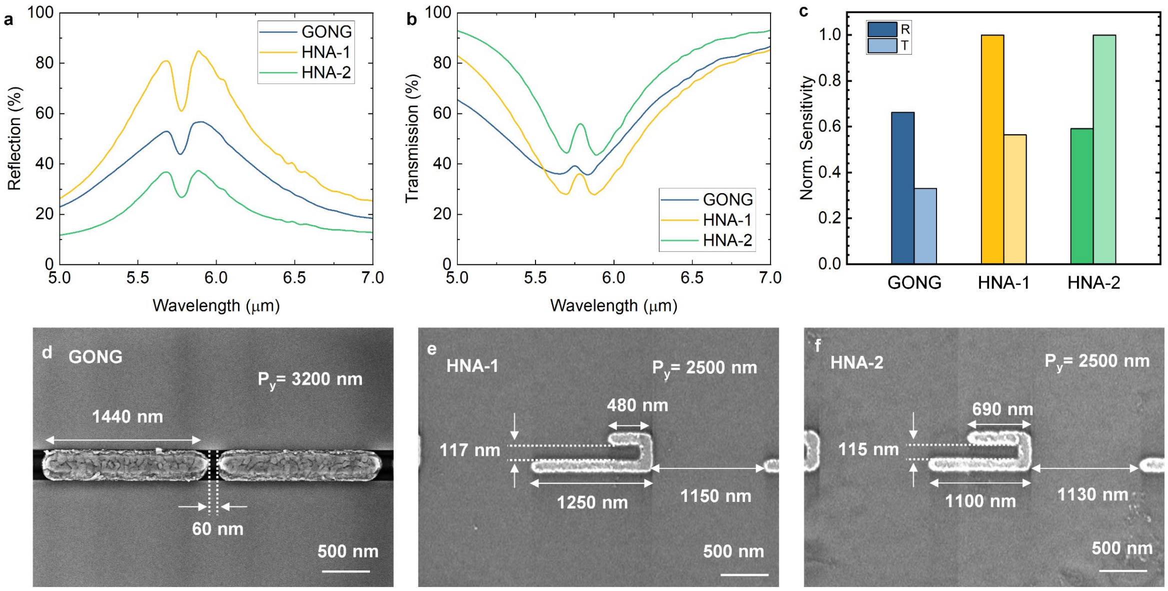

Fig. S7 a,b PMMA sensing testing results of grating order nanogap (GONG) designs and HNA designs in reflection (**a**) and transmission mode (**b**). **c** the extracted normalized sensitivity of different sensors. HNA-1 shows an improvement of 50% in reflection mode and HNA-2 shows an improvement of 200% in transmission mode. By engineering the loss of HNA from geometric design, the sensing performance can be improved without the introduction of nanogap. **d-f** SEM image of fabricated GONG sensor with the nanogap of 60 nm(**d**), HNA-1 (**e**), and HNA-2 (**f**) sensors.

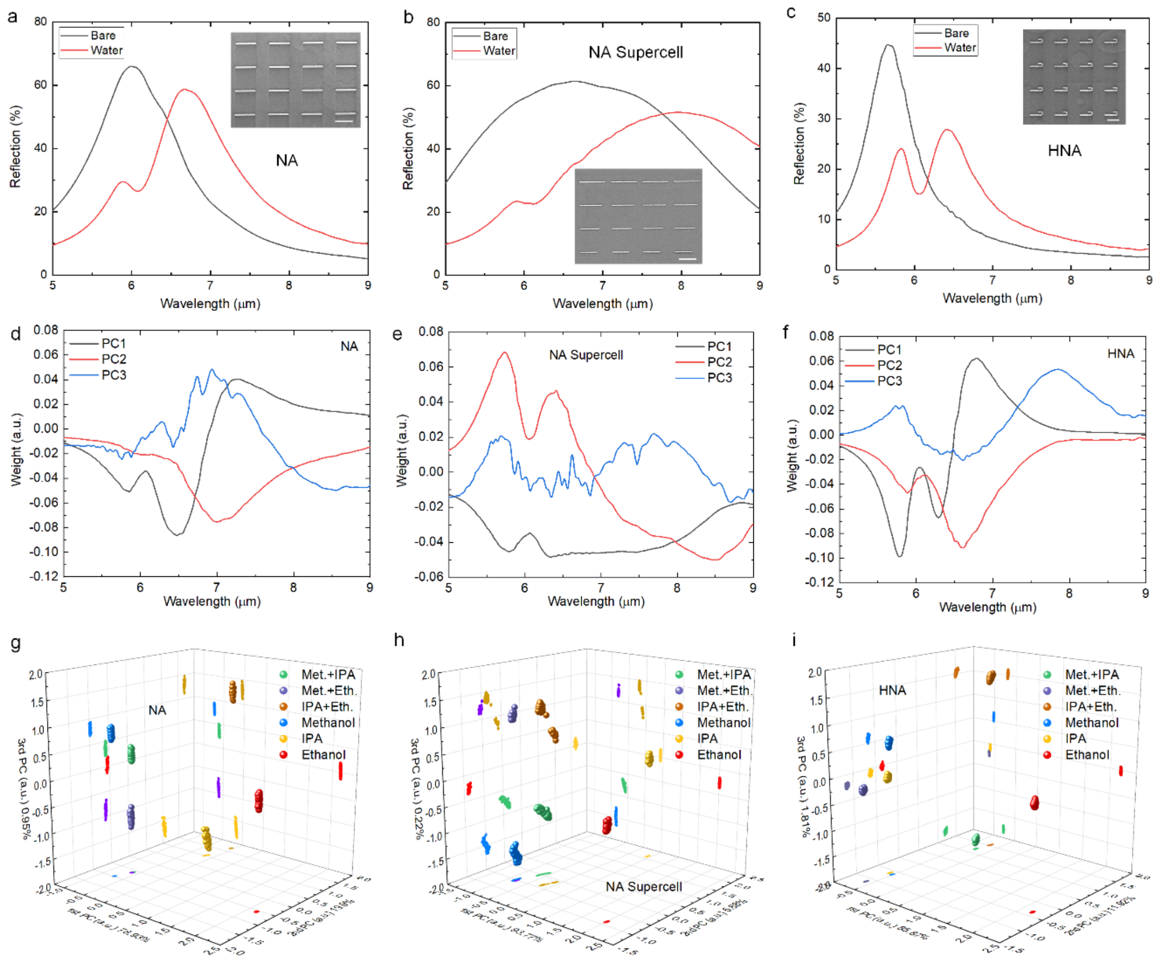

Fig. S8 The reference data for different types of nanoantenna devices using machine learning for recognition of alcoholic molecules. a-c, the spectra of NA, NA supercell, and HNA tested by the bare antenna and in water. d-f, the PCA-processed spectra for each device. g-i, the data distribution in PC space.

Note S7: Molecular identification using deep neural network

Our DNN dataset comprises 300×1272 data points and we randomly split these data, in which 80% for training data set, 20% for testing data set. The proposed DNN model is built using the Sequential model of Python's Keras frame. Each fully connected layer followed with ReLu as activation function and the final model was compiled by 'categorical_corssentropy' as the loss function and Adam as the optimizer. We set the batch size as 20. In the 800 epochs, the loss between prediction and ground truth shows excellent convergence in both of training and testing set, and there is no overfitting occurring either. (**Fig. S9c**) Finally, 100% identification accuracy is obtained for alcohols identification shown in **Fig. S9d,e**.

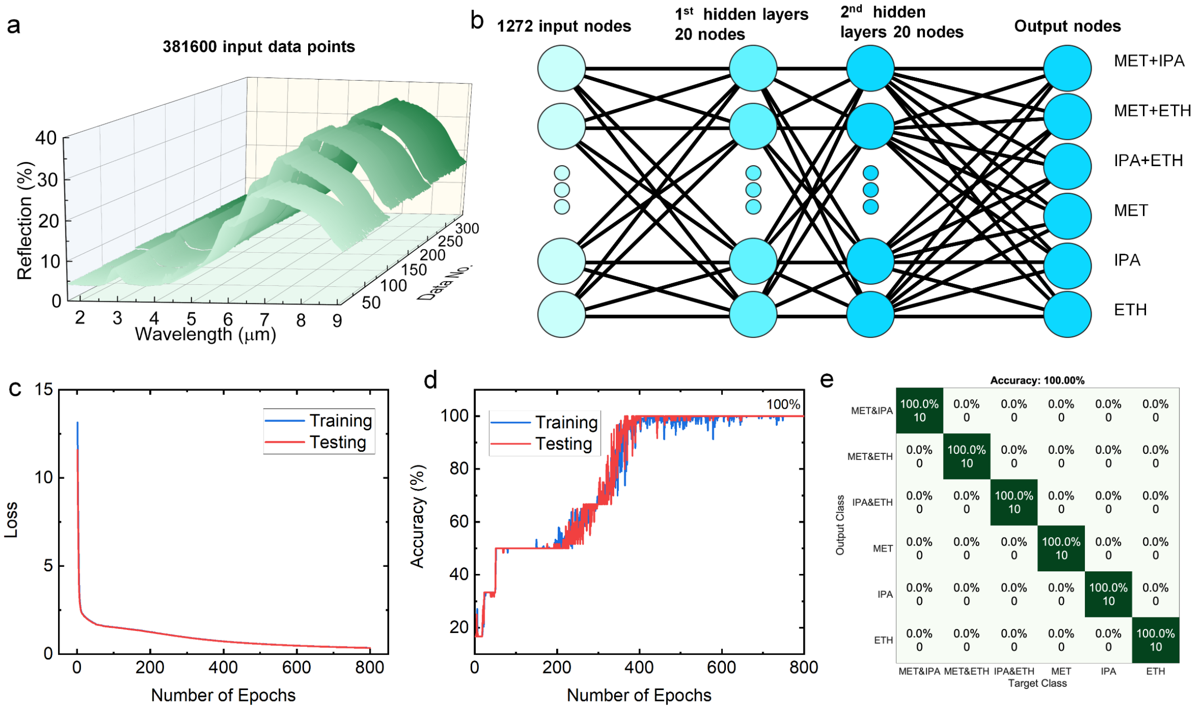

Fig. S9 Alcohol identification from microfluidics integrated WMHNA using DNN. **a** The spectral data of different combinations of 1% alcohol solvent. **b** The proposed DNN model with two hidden layers and 20 nodes for identification of 6 different analytes. **c,d** the loss and accuracy of the DNN model at the different number of epochs. **e** The confusion map of final results for alcohol identification.

References:

1. Dong, L. *et al.* Nanogapped Au Antennas for Ultrasensitive Surface-Enhanced Infrared Absorption Spectroscopy. *Nano Lett.* **17**, 5768–5774 (2017).
2. Yoo, D. *et al.* High-Contrast Infrared Absorption Spectroscopy via Mass-Produced Coaxial Zero-Mode Resonators with Sub-10 nm Gaps. *Nano Lett.* **18**, 1930–1936 (2018).
3. Rodrigo, D. *et al.* Mid-infrared plasmonic biosensing with graphene. *Science (80-.).* **349**, 165–168 (2015).
4. Zhu, Y. *et al.* Optical conductivity-based ultrasensitive mid-infrared biosensing on a hybrid metasurface. *Light Sci. Appl.* **7**, 67 (2018).
5. Xu, J. *et al.* Nanometer-Scale Heterogeneous Interfacial Sapphire Wafer Bonding for Enabling Plasmonic-Enhanced Nanofluidic Mid-Infrared Spectroscopy. *ACS Nano* **14**, 12159–12172 (2020).
6. Hwang, I. *et al.* Ultrasensitive Molecule Detection Based on Infrared Metamaterial Absorber with Vertical Nanogap. *Small Methods* **5**, 2100277 (2021).
7. John-Herpin, A., Kavungal, D., Mücke, L. & Altug, H. Infrared Metasurface Augmented by Deep Learning for Monitoring Dynamics between All Major Classes of Biomolecules. *Adv. Mater.* **33**, 2006054 (2021).
8. Miao, X., Yan, L., Wu, Y. & Liu, P. Q. High-sensitivity nanophotonic sensors with passive trapping of analyte molecules in hot spots. *Light Sci. Appl.* **10**, 5 (2021).
9. Nuñez, J., Boersma, A., Grand, J., Mintova, S. & Sciacca, B. Thin Functional Zeolite Layer Supported on Infrared Resonant Nano-Antennas for the Detection of Benzene Traces. *Adv. Funct. Mater.* **31**, 2101623 (2021).
10. Zhou, H. *et al.* Metal–Organic Framework-Surface-Enhanced Infrared Absorption Platform

Enables Simultaneous On-Chip Sensing of Greenhouse Gases. *Adv. Sci.* **7**, 2001173 (2020).

11. Li, D. *et al.* Multifunctional Chemical Sensing Platform Based on Dual-Resonant Infrared Plasmonic Perfect Absorber for On-Chip Detection of Poly(ethyl cyanoacrylate). *Adv. Sci.* **8**, 2101879 (2021).

12. Hui, X. *et al.* Infrared Plasmonic Biosensor with Tetrahedral DNA Nanostructure as Carriers for Label-Free and Ultrasensitive Detection of miR-155. *Adv. Sci.* **8**, 2100583 (2021).

13. Wu, C. *et al.* Fano-resonant asymmetric metamaterials for ultrasensitive spectroscopy and identification of molecular monolayers. *Nat. Mater.* **11**, 69–75 (2012).

14. Adato, R. & Altug, H. In-situ ultra-sensitive infrared absorption spectroscopy of biomolecule interactions in real time with plasmonic nanoantennas. *Nat. Commun.* **4**, 2154 (2013).

15. Fan, S., Suh, W. & Joannopoulos, J. D. Temporal coupled-mode theory for the Fano resonance in optical resonators. *J. Opt. Soc. Am. A* **20**, 569 (2003).
